# Natural progression of glioma enhances functional connection with the cerebral cortex through synaptogenesis

**DOI:** 10.1016/j.nicl.2026.103942

**Published:** 2026-01-04

**Authors:** Jiacheng Lai, Yan Bai, Hongbo Bao, Shuai Wu, Xinyu Wang, Xia Liang, Peng Liang

**Affiliations:** a Department of Neurosurgery, Harbin Medical University Cancer Hospital, Harbin, China; b Department of Life Science and Medicine, Harbin Institute of Technology, Harbin 150001, China; c Research Center for Social Computing and Information Retrieval, Harbin Institute of Technology, Harbin 150001, China; d Department of Neurosurgery, BeijingTiantan Hospital, Capital Medical University, Beijing, China; e Frontier Science Center for Interaction between Space Environment and Matter, Harbin Institute of Technology, Harbin 150001, China

**Keywords:** Glioma, Natural progression, Cancer neuroscience, Resting-state functional magnetic resonance imaging, Single-cell RNA sequencing

## Abstract

**Objectives:**

Understanding the progression mechanisms of glioma holds significant implications for improving clinical management. However, the natural progression patterns of glioma remain poorly understood due to the lack of longitudinal clinical samples from untreated patients.

**Materials and methods:**

In this study, we systematically explored the natural progression trajectory of glioma by combining functional magnetic resonance imaging (fMRI) analysis of 24 rare multifocal glioma patients with bioinformatic analysis of single-cell RNA sequencing (scRNA-seq) data obtained from tumor samples of glioma mouse with early, mid, and endpoint lesions.

**Results:**

We discovered that larger tumors in multifocal gliomas exhibit stronger functional connectivity with the cerebral cortex and higher degree centrality within brain networks. ScRNA-seq of longitudinal mouse glioma samples revealed progressive activation of synaptic organization and associated regulatory pathways during the natural progression of glioma.

**Conclusion:**

Our multimodal, cross-scale study demonstrates that the natural progression pattern of glioma macroscopically manifests as functional hyperconnectivity with the cerebral cortex, which is supported by microscale molecular programs driving synaptogenesis. These findings elucidate the characteristics and mechanisms underlying glioma natural progression.

## Highlights

• Multifocal glioma patients and mouse models help address the lack of longitudinal data in untreated patients.
• In multifocal gliomas, larger tumors exhibit stronger cortical functional connectivity and higher degree centrality.
• During glioma progression, synaptic organization and associated pathways are progressively activated.

## Graphical abstract

**Figure:**
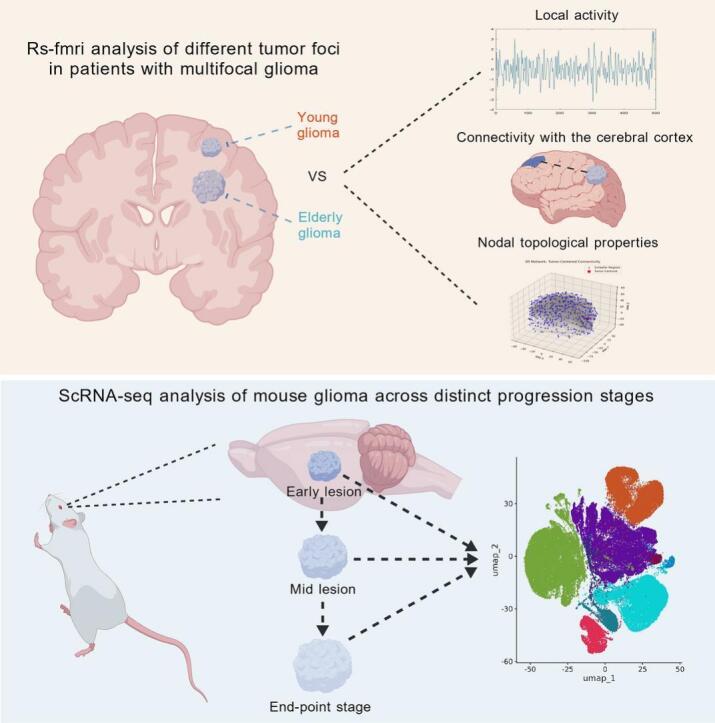

## 1 Introduction

Glioma is the most common primary malignant intracranial tumor in adults, characterized by high disability and mortality rates (Louis et al., 2021). *Isocitrate dehydrogenase* (*IDH*)-wildtype glioblastoma represents the most aggressive molecular subtype, with a median survival time of approximately 14.4 months (Schaff and Mellinghoff, 2023). Importantly, according to the 2021 WHO classification, *IDH*-wildtype diffuse astrocytic tumors that appear radiologically non-enhancing or histologically ‘low-grade’ may already correspond to molecular glioblastoma (Louis et al., 2021), highlighting the limitation of conventional grading and the clinical relevance of molecular stratification. Despite advances in cancer therapy over the past two decades, glioma prognosis remains dismal due to its complex progression mechanisms (Chen et al., 2012, Laug et al., Jul 2018). Therefore, deciphering glioma progression patterns is crucial for improving treatment. While the development of capabilities such as proliferation, invasion, and chemoresistance has been extensively studied in gliomas, the natural progression trajectory and underlying mechanisms of these tumors remain poorly understood, likely due to the scarcity of longitudinal samples and clinical data from patients.

Confronting this challenge, the vast majority of prior studies have inferred glioma progression by comparing primary and recurrent tumors (Wang et al., Jul 2016, Barthel et al., Dec 2019, Kim et al., Mar 2015, Gerstung et al., Feb 2020), but this approach cannot distinguish natural tumor progression from treatment-induced effects. For example, temozolomide chemotherapy not only kills glioma cells but also enhances leucine metabolism, thereby inducing radioresistance in tumor cells (Zang et al., 2024). Moreover, radiotherapy and chemotherapy significantly alter the tumor microenvironment and can induce tumor cells into a senescent-like state. Although these cells temporarily cease proliferation, they can stimulate non-senescent tumor cells to promote recurrence and enhance the invasiveness and drug resistance of recurrent tumors (Riviere-Cazaux et al., 2023). Multifocal gliomas offer a unique model for addressing this challenge. These patients, representing 0.5 %-20 % of all glioma cases, present with multiple independent tumor lesion within the brain, which result from tumor dissemination or growth along defined pathways such as white matter tracts, the ventricular system, or local spread forming satellite lesions (Giannopoulos and Kyritsis, 2010, Lasocki et al., Sep 2016). Different tumor lesions in the same patient share a common clonal origin, represent distinct “age” stages (Abou-El-Ardat et al., 2017), and remain unaffected by therapeutic interventions. Wu et al. identified a potential evolutionary order between two lesions in multifocal gliomas, confirming that the first lesion is older than the second, with the latter exhibiting a tendency to develop phenotypes resembling tumor cells in the first lesion (Wu et al., 2022). Additionally, employing mouse glioma models can overcome the clinical and ethical challenges associated with longitudinal observation and sampling of untreated patient tumors. Hamed et al. utilized conditional gene deletion and lineage tracing in glioma mouse models combined with serial magnetic resonance imaging to track tumor progression trajectories, revealing that processes resembling injury response are activated during early tumorigenesis (Hamed et al., Feb 2025). These findings provide a theoretical foundation for the present study to investigate the natural progression patterns of gliomas using both multifocal gliomas patients and glioma mouse models.

Glioma progression has been demonstrated to be associated with multiple contributing factors, with previous investigations primarily focusing on the influences of tumor genetic characteristics and the local microenvironment (Quail and Joyce, 2017, Lin et al., 2024). However, recent research in the field of cancer neuroscience has demonstrated that the nervous system serves as a critical regulator of glioma development (Mancusi and Monje, 2023, Winkler et al., 2023). Neuronal activity participates in modulating glioma initiation and progression (Venkatesh et al., 2015, Pan et al., Jun 2021, Venkatesh et al., 2017), while gliomas, in turn, exploit the structural and functional components of the nervous system to integrate into brain networks, influencing cerebral function and promoting their own growth (Venkatesh et al., Sep 2019, Venkataramani et al., Sep 2019). Even remote neuronal activities in the hemisphere contralateral to the tumor can propagate signals through structural connections to facilitate tumor progression (Huang-Hobbs et al., Jul 2023). These findings suggest that the natural progression pattern of glioma may not only be associated with tumor-intrinsic characteristics but could also involve interactions between tumors and the nervous system. Resting-state functional magnetic resonance imaging (rs-fMRI) can quantifie local activity characteristics in brain regions and functional connectivity (FC) between different areas by measuring blood oxygen level-dependent signal fluctuations (Chen and Glover, Sep 2015). Rs-fMRI-based studies have established the clinical significance of amplitude of low-frequency fluctuation (ALFF), fractional ALFF (fALFF), regional homogeneity (ReHo), and tumor-to-brain FC for glioma diagnosis, classification, and prognosis prediction (Agarwal et al., Aug 2017, Gupta et al., Jun 2018, Boyer et al., Aug 2016, Daniel et al., 2021). However, their changes during the natural progression of gliomas and the underlying molecular biological mechanisms remain unclear.

In this study, we conducted a multimodal and cross-scale systematic investigation of the natural progression of gliomas by integrating advanced neuroimaging techniques with single-cell sequencing technology. First, for each patient with multifocal glioma, the two tumor lesions were stratified into larger and smaller groups based on their relative volumes. The larger tumor was regarded as representing a later stage of progression, whereas the smaller tumor represented an earlier stage. Using rs-fMRI, we performed paired comparisons of tumor activity characteristics, alterations in FC between tumors and the cerebral cortex, and nodal topological properties to investigate evolving patterns during glioma natural progression. Subsequently, we analyzed of single-cell RNA sequencing (scRNA-seq) data from mouse glioma samples across distinct progression stages to further elucidate the molecular mechanisms underlying glioma natural progression. Our study reveals that gliomas exhibit enhanced FC with the cerebral cortex and increased integration into brain networks during natural progression, which is supported by microscale molecular programs driving synaptogenesis.

## 2 Materials and methods

### 2.1 Patients

**Fig. 1:**
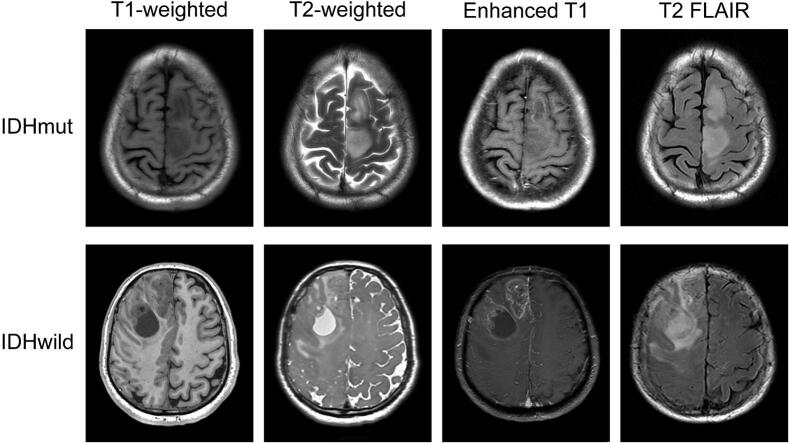
**Multiple sequence MRI examples of IDH mutant and IDH wild type multifocal gliomas.** IDHmut, IDH mutant type. IDHwild, IDH wild type.

This single-center retrospective cohort study was approved by the Institutional Review Board of Harbin Medical University Cancer Hospital (KY2021-42), which requires no specific patients’ informed consent when retrospective patients’ data are analyzed, and conducted in accordance with the Declaration of Helsinki. This study enrolled 24 patients with pathologically confirmed multifocal gliomas, all of whom exhibited two independent lesions and met at least one of the following MRI-based criteria (Lasocki et al., Sep 2016): (a) presence of hyperintense connecting signals between lesions on fluid attenuated inversion recovery (FLAIR) sequences; (b) adjacency of both lesions to the ventricular system; or (c) formation of satellite lesions around the primary tumor. Examples of multifocal gliomas with different *IDH* mutation statuses are presented in Fig. 1. All patients underwent preoperative rs-fMRI examinations. Pathological diagnosis was established according to the World Health Organization 2021 classification of central nervous system tumors (Louis et al., 2021). Exclusion criteria included: (a) prior brain surgery or radiotherapy; (b) coexisting neurological disorders or other tumor types; (c) incomplete or poor-quality imaging data.

### 2.2 Image acquisition

Preoperative MRI was performed on a GE Architect 3.0 T scanner with the following protocols: (a) High-resolution T1/T2-weighted images (TR/TE = 2641 ms/2.51 ms, inversion time = 1116 ms, flip angle = 8°, field of view = 240 mm × 240 mm, voxel size 0.5 × 0.47 × 0.47 mm^3^, number of slices = 372). (b) Resting-state fMRI using gradient-echo EPI (TR/TE = 1600 ms/30 ms, flip angle = 90°, acquisition matrix = 64 × 64 pixels, field of view = 240 mm × 240 mm, number of slices = 65, slice thickness = 3 mm, slice gap = 0, voxel size = 3 × 3 × 3 mm^3^, with a total of 500 whole-brain images acquired).

### 2.3 Tumor segmentation and fMRI processing

With reference to T1-enhanced and T2 FLAIR images, two experienced neuroradiologists independently delineated the tumor boundaries manually on high-resolution T2-weighted images using 3D Slicer v4.13.0 (https://www.slicer.org/) (Fedorov et al., Nov 2012). In cases where the volumetric difference exceeded 5 %, a consensus review was conducted to resolve discrepancies. The resulting binary tumor masks underwent nonlinear spatial normalization to MNI152 standard space. Functional MRI processing was implemented through fMRIprep (Esteban et al., Jan 2019), incorporating removal of initial five volumes to account for magnetic stabilization, slice-time correction, motion realignment, field distortion correction, and final registration to MNI space. Specifically, fMRIprep applied symmetric normalization based susceptibility distortion correction to address magnetic field inhomogeneities. This method estimates distortion by leveraging spatial correspondence between T1-weighted structural and fMRI functional images, with particular attention to phase-encoding direction distortions, and then corrects the fMRI data accordingly. During registration, functional images were first aligned to the individual’s T1-weighted structural image. The T1-weighted image was then nonlinearly registered to standard space, with quality control implemented to ensure accuracy. Finally, the tumor region outlined on T2-weighted images was co-registered to the same standard space via a two-step transformation. To establish a “healthy” control group, this study flipped the tumor mask towards the contralateral hemisphere to obtain a symmetrical region mask as a control. Following registration of each tumor ROI, we used the Yeo 7-network atlas as a template to determine the number of distinct resting-state networks intersected by the ROI (Yeo et al., Sep 2011). Subsequently, the Nilearn package (https://nilearn.github.io) (Abraham et al., 2014) was used to remove head motion, white matter and cerebrospinal fluid covariates, smooth with a 5 mm FWHM Gaussian kernel and apply band-pass filtering (0.01–0.1 Hz). To control for potential head-motion effects, we computed the mean framewise displacement (FD) and the mean derivative of root mean square variance over voxels (DVARS) for each patient (Power et al., 2012, Fair et al., 2012). Two patients were excluded because their mean FD exceeded 0.2  mm and their mean DVARS exceeded 20. Data from the remaining 24 participants were carried forward for further analysis (Fig. S1). After extracting the time series of each glioma ROI, we computed its temporal signal-to-noise ratio (tSNR) by dividing the mean time series of the ROI by the standard deviation of the BOLD run (Bodurka et al., 2007).

### 2.4 Glioma activity characteristics

Glioma activity metrics include ALFF, fALFF, and ReHo. Local activity was quantified through ALFF computed as integrated power spectral density within the 0.01–0.1 Hz band (Zang et al., Mar 2007), with fALFF representing the ratio of this low-frequency power to total power across 0–0.25 Hz (Zou et al., 2008). ReHo was derived using Kendall's coefficient of concordance across 27-voxel neighborhoods (Zang et al., May 2004). All metrics underwent z-score normalization within subjects. We calculated ALFF, fALFF, and ReHo values within both tumor masks and control mask regions, followed by interlesional comparisons of these metrics and their differential values relative to controls among patients.

### 2.5 FC analysis

The Schaefer 400 cortical parcellation atlas was used to segment the cerebral cortex into 400 distinct regions (Schaefer et al., 2018). For each patient's analysis, tumor masks were subtracted from the brain parcellation to exclude tumor effects. Seed-based correlation analysis was performed using each tumor mask as a region of interest (ROI): Pearson's r correlation coefficients were computed between the mean time series of tumor ROIs and the mean time series of each cortical parcel, followed by Fisher z-transformation of correlation coefficients. The correlations between tumor ROIs and brain regions underwent Bonferroni correction to identify cortical parcels showing significant associations with tumors, followed by computation of Euclidean distances between tumor centroids and centroids of significantly related regions. FC between control mask regions and cortical parcels was similarly analyzed to identify significant connections and their spatial distances, enabling interlesional comparisons of these metrics and their differential values relative to controls.

### 2.6 Network analysis

To elucidate the functional relationship between glioma natural progression and the nervous system, we conducted network-based analyses of tumors and cerebral cortex. Functional networks were constructed by treating each tumor ROI (or its contralateral control ROI) and the 400 cortical parcels as network nodes. FC was computed between every pair of nodes, and edges were retained according to a sparsity threshold. The range of sparsity values was selected to ensure that no isolated nodes remained in the functional network of any participant while also maintaining a small-world index >1.1 (Huang et al., 2024). Based on these criteria, a sparsity range of 0.15–0.40 was used, examined at intervals of 0.05. At each sparsity level, the topological properties (degree centrality, betweenness centrality, closeness centrality, and clustering coefficient) of each ROI node were calculated using NetworkX, followed by within-subject comparisons between the lesions.

### 2.7 Bioinformatics analysis

ScRNA-seq data of mouse gliomas across distinct progression stages were obtained from the Gene Expression Omnibus (GEO) database (accession number: GSE278511). The mouse glioma model was established by Hamed et al., with fresh tumor tissues collected at distinct stages defined by MRI criteria (Hamed et al., Feb 2025). The early stage was defined by the detection of subtle abnormalities in the mouse brain on FLAIR; the mid stage was characterized by further tumor enlargement in the absence of clinical symptoms; and the endpoint stage corresponded to continued tumor growth accompanied by signs of elevated intracranial pressure or focal neurological deficits (Hamed et al., Feb 2025). Following single-cell RNA sequencing of tumor tissues across these stages, Hamed et al. performed quality control, data integration, dimensionality reduction, and clustering, culminating in the annotation of diverse cell populations (Hamed et al., Feb 2025). From these annotated datasets, we extracted malignant cells from early, mid, and endpoint stages for subsequent downstream analysis. Pseudotime trajectory inference was performed on these stage-specific malignant cell populations using the Monocle3 package in R. Gene Ontology (GO) enrichment and Gene Set Enrichment Analysis (GSEA) of differentially expressed genes among glioma cells across stages were conducted using the clusterProfiler package. Bulk transcriptome data from glioma patients were sourced from the mRNAseq_325 dataset within the Chinese Glioma Genome Atlas (CGGA) database. Kaplan-Meier survival analysis was employed to evaluate the correlation between key differentially expressed genes and patient prognosis.

### 2.8 Statistical analysis

Comparisons of tumor activity characteristics, functional connectivity with the cerebral cortex, and nodal topological properties in brain networks between the two tumor lesion groups were conducted as follows. The normality of distribution for inter-group differences was primarily evaluated using the Shapiro-Wilk test. For differences conforming to a normal distribution, paired t-tests were applied; otherwise, the Wilcoxon signed-rank test was used. Since functional brain regions can be partially preserved within gliomas (Cui et al., Mar 2022), the spatial location differences of distinct tumor lesion may influence tumor characteristics. Following the approach described by Jutten et al. (Jutten et al., 2025), we selected symmetric regions in the contralateral hemisphere as internal controls. Following direct comparisons or correlation analyses using tumor lesion, we further performed analyses using the differences between tumor lesion and their contralateral control regions to account for spatial heterogeneity. A result was considered positive only when statistical significance was achieved in both analytical approaches. A linear mixed-effects model (LMM) was employed to control for potential confounding. In the model, the significantly different features served as dependent variables, patient ID was included as a random intercept, and tumor group, volume, tSNR, and the number of intersecting resting-state networks were incorporated as fixed effects. This allowed us to evaluate whether the between-group differences remained significant after adjusting for these covariates. In each section of the analysis, Bonferroni correction was used together to correct the multiple comparison results for both analytical approaches. All results were visualized using ggplot2 and enrichplot, while statistical analyses were performed in R (v4.3.2). A threshold of *p* < 0.05 was considered statistically significant.

## 3 Results

### 3.1 Patient characteristics

Twenty-four subjects diagnosed with multifocal gliomas were enrolled in this study (Table 1), with a mean age of 51.4 ± 8.0 years and a male predominance (2/3 of the cohort). According to WHO grading, 12 patients had low-grade gliomas and 12 had high-grade gliomas. Based on the WHO 2021 Classification of Central Nervous System Tumors, 19 patients harbored *IDH*-mutant tumors and 5 patients had *IDH*-wildtype tumors. The pathological subtypes among these patients included astrocytoma (n = 11), oligodendroglioma (n = 8), and glioblastoma (n = 5). All patients exhibited two distinct tumor lesions, which were grouped by volume: the larger tumor group had a mean volume of 78.6 ± 33.7 cm^3^, while the smaller tumor group measured 24.9 ± 15.3 cm^3^.

**Table 1:** Baseline characteristics of multifocal glioma patients.

**Characteristic**	**Multifocal glioma** **(n = 24)**
**Age (± SD), years**	51.4 ± 8.0
**Sex, n (%)**	
Male	16 (66.7 %)
Female	8 (33.3 %)

**WHO grade, n (%)**	
Ⅱ	12 (50.0 %)
Ⅲ	7 (29.2 %)
Ⅳ	5 (20.8 %)

**IDH mutation state, n (%)**	
IDH mutant type	19 (79.2 %)
IDH wild type	5 (20.8 %)

**Tumor histology, n (%)**	
Astrocytoma	11 (45.8 %)
Oligodendroglioma	8 (33.3 %)
Glioblastoma	5 (20.8 %)

**Tumor volume (±SD), cm^3^**	
Larger tumor	78.6 ± 33.7
Smaller tumor	24.9 ± 15.3

### 3.2 No significant differences in activity between different tumor lesions

**Fig. 2:**
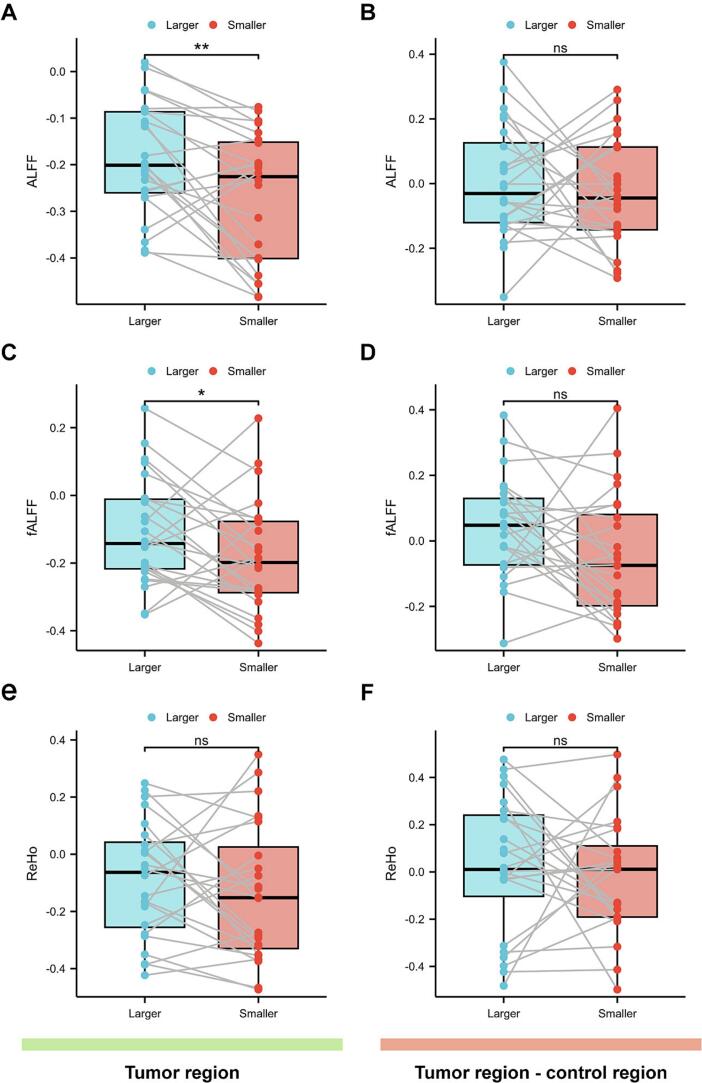
**Comparison of local tumor activity characteristics in multifocal gliomas.** Paired comparisons were performed to assess differences in ALFF (A), fALFF (C), and ReHo values (E) between larger and smaller tumor foci in multifocal gliomas, followed by intergroup comparisons of the differences between tumor and control regions (B, D, F).

We initially calculated activity metrics, including ALFF, fALFF, and ReHo, for each tumor lesion and their contralateral hemispheric control regions. The results revealed that ALFF values were significantly higher in larger tumors compared to smaller tumors in multifocal gliomas (*p* = 0.003, *Cohen's d* = 0.676, Fig. 2A). To account for potential spatial localization effects, we further compared the ALFF differences between tumor regions and their corresponding control regions, which showed no significant intergroup differences (*p* = 0.520, *Cohen's d* = 0.133, Fig. 2B). These findings suggest that the observed ALFF variations between multifocal glioma lesions are not tumor-specific. Although the fALFF of the larger tumor is higher than that of the smaller tumor (*p* = 0.016, *Wilcoxon r* = 0.780*,* Fig. 2C), its significance failed to be Bonferroni-corrected (the threshold for *p* is 0.008), and the difference was not expected to be significant after subtracting the control (*p* = 0.059, *Cohen's d* = 0.406, Fig. 2D). In contrast, ReHo showed no significant difference between tumor size groups, whether analyzing the original values or the differences relative to contralateral control regions (*p* = 0.322, *Cohen's d* = 0.207, Fig. 2E; *p* = 0.567, *Cohen's d* = 0.119, Fig. 2F).

### 3.3 Larger tumor lesions exhibit stronger cortical connectivity

**Fig. 3:**
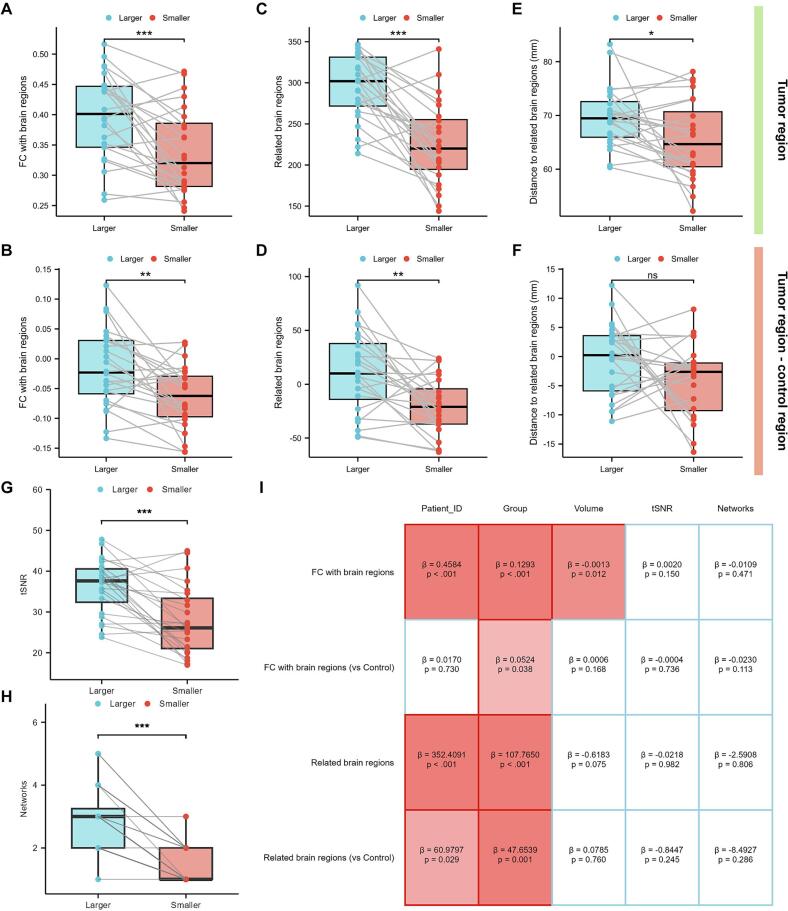
**Differences in glioma-cortical connectivity.** Paired comparisons were conducted to evaluate differences between larger and smaller tumor in multifocal gliomas regarding: (A) mean FC with the cerebral cortex, (C) number of significantly related brain regions, and (E) spatial distances between tumors and their significantly related brain regions. Subsequently, inter-group comparisons were conducted to assess the differences in tumor changes compared to the control area between the two groups (B, D, F). Comparison of tSNR (G) and the number of intersected brain networks (H) between larger and smaller tumor groups. (I) After controlling for confounding factors such as tumor volume, tSNR, and the number of intersected brain networks in the LMMs, significant differences between the two groups of tumors still persisted. FC, functional connectivity. LMM, Linear mixed-effects model. tSNR, temporal signal-to-noise ratio.

To evaluate the association between different tumor lesions and the cerebral cortex in multifocal gliomas, we parcellated the cortex into 400 regions and compared the mean FC between lesions and brain regions, the number of related brain regions, and their distance from related brain regions. We found that the mean FC between larger tumors and the cortex was significantly stronger than that of smaller tumors (*p* < 0.001, *Cohen's d* = 0.781, Fig. 3A). This difference remained significant after accounting for tumor location by comparing connectivity differences between tumor and contralateral control regions, with larger tumors still exhibiting higher cortical connectivity (*p* = 0.003, *Cohen's d* = 0.669, Fig. 3B). To further characterize glioma-cortical connectivity changes, we performed Bonferroni-corrected Pearson correlation analysis between tumors and cortical regions, identifying significantly correlated regions. Larger tumors showed a greater number of significantly related brain regions compared to smaller tumors (*p* < 0.001, *Cohen's d* = 1.152, Fig. 3C), and this difference persisted after controlling for spatial effects (*p* = 0.001, *Cohen's d* = 0.763, Fig. 3D). These results suggest that glioma progression drives widespread enhancement of FC across the cortex. Additionally, larger tumors were located farther from their related brain regions than smaller tumors (*p* = 0.013, *Cohen's d* = 0.547, Fig. 3E), although this significance did not survive Bonferroni correction (the threshold for *p* is 0.008). Moreover, the inter-group difference in distance relative to control regions showed no statistical significance (*p* = 0.084, *Cohen's d* = 0.369, Fig. 3F).

However, tSNR can scale with ROI size and may spuriously elevate FC estimates. We compared the tSNR between the two groups of tumors and found that the larger-tumor group exhibited significantly higher tSNR than the smaller-tumor group (*p* < 0.001, *Cohen's d* = 1.014, Fig. 3G). Furthermore, larger tumor ROIs may span more resting-state networks, which could influence FC estimates. A between-group comparison confirmed that larger tumors intersect a greater number of resting-state networks than smaller tumors (*p* < 0.001, *Wilcoxon r* = 0.832, Fig. 3H). Given that the two tumor groups differed significantly in volume, tSNR, and the number of intersecting resting state networks, it was unclear whether the observed differences in cortical FC reflected true progression-related changes or were confounded by these factors. We therefore performed an LMM to account for these potential confounds, as shown in Fig. 3I. After controlling for all covariates, FC remained significantly higher in the larger tumor group compared with the smaller tumor group (*β* = 0.129, *p* < 0.001). Among the covariates, tumor volume showed a significant negative association with FC (*β* = -0.001, *p* = 0.012), whereas tSNR (*β* = 0.002, *p* = 0.150) and the number of intersecting networks (*β* = -0.011, *p* = 0.471) did not reach significance. When FC was examined after subtracting the contralateral control region, the group difference persisted (*β* = 0.052, *p* = 0.038), while none of the covariates, including tumor volume (*β* = 0.001, *p* = 0.168), tSNR (*β* = -0.0004, *p* = 0.736), and the number of intersecting networks (*β* = -0.023, *p* = 0.113), were significant. Moreover, the larger tumor group exhibited a significantly greater number of related brain regions than the smaller tumor group (*β* = 107.765, *p* < 0.001). This difference remained significant after subtracting the contralateral control (*β* = 47.654, *p* = 0.001), and no other covariates showed a significant effect.

### 3.4 Larger tumor lesions exhibit higher degree centrality in brain networks

**Fig. 4:**
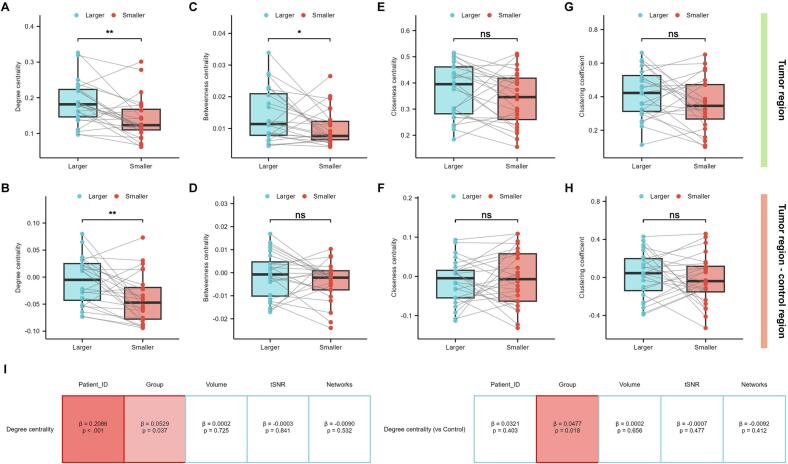
**Differences in nodal topological properties of tumors within brain networks.** By incorporating either gliomas or control regions as nodes in brain network construction, paired comparisons were performed between larger and smaller tumors for: (A) degree centrality, (C) betweenness centrality, (E) closeness centrality, and (G) clustering coefficient. Intergroup comparisons were subsequently conducted for differences between tumor and control regions (B, D, F, H). (I) Further analysis using LMMs, adjusted for confounding factors including tumor volume, tSNR, and the number of intersected resting-state networks, was conducted to examine whether differences in degree centrality between the two tumor groups remained significant. LMM, Linear mixed-effects model. tSNR, temporal signal-to-noise ratio.

The human brain operates through coordinated information transfer across neural networks, with cognitive functions and behavioral outputs relying on dynamic network interactions (Varela et al., Apr 2001). In this study, we incorporated gliomas as network nodes to assess their topological properties within whole-brain networks. At a sparsity threshold of 0.15, a comparative analysis of the topological properties between tumor lesions revealed that larger tumors exhibited significantly higher degree centrality than smaller tumors (*p* = 0.002, *Cohen's d* = 0.707, Fig. 4A). This difference remained significant after subtracting the values from the control regions (*p* = 0.004, *Cohen's d* = 0.653, Fig. 4B). Larger tumors also showed higher betweenness centrality compared with smaller tumors (*p* = 0.050, *Cohen's d* = 0.423, Fig. 4C); however, this difference did not survive Bonferroni correction and became non-significant after subtraction of the control (*p* = 0.228, *Cohen's d* = 0.253, Fig. 4D). In contrast, neither closeness centrality nor clustering coefficient differed significantly between groups, either in raw values or after controlling for spatial effects (*p* = 0.177, *Cohen's d* = 0.284, Fig. 4E; *p* = 0.625, *Cohen's d* = -0.101, Fig. 4F; *p* = 0.117, *Cohen's d* = 0.333, Fig. 4G; *p* = 0.920, *Cohen's d* = 0.333, Fig. 4H). The results remained stable different thresholds ranging from 0.15 to 0.4: the degree centrality of larger tumors was consistently and significantly higher than that of smaller tumors, both in raw values and after subtracting the contralateral control (Fig. S2). To further account for potential confounding by tumor volume, tSNR, and the number of intersecting resting-state networks, we applied a LMM. The model confirmed that degree centrality remained significantly higher in larger tumors than in smaller tumors, both for raw values and after control subtraction (*β* = 0.053, *p* = 0.037; *β* = 0.048, *p* = 0.018, Fig. 4I).

### 3.5 Progressive activation of synaptic organization pathway in glioma cells

**Fig. 5:**
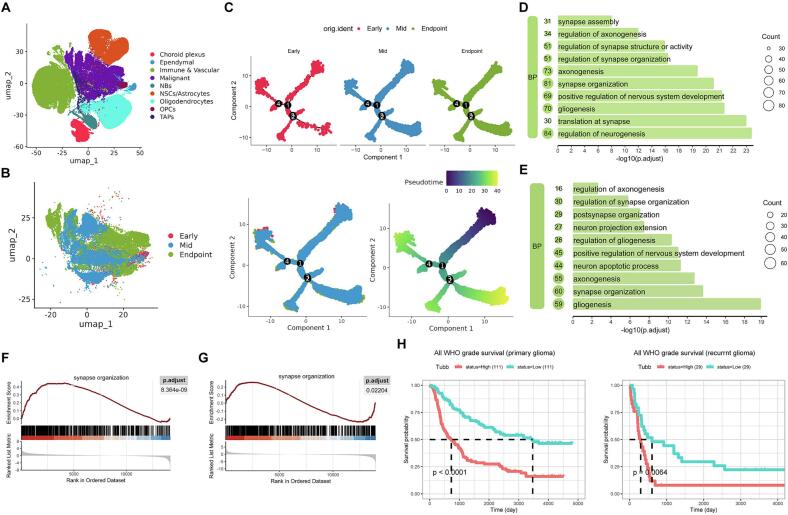
**Transcriptomic remodeling of glioma cells during natural progression.** (A) UMAP projection of single-cell RNA sequencing data from mouse gliomas at early, mid, and endpoint stages, illustrating 9 distinct cellular clusters. Each point represents an individual cell, colored by cluster identity. (B) UMAP visualization of malignant cells isolated from gliomas across the 3 stages, colored by progression phase. (C) Differentiation trajectory of malignant cells reconstructed for each stage, followed by integrated pseudotemporal ordering to validate the chronological progression of malignant states. (D, E) GO biological process enrichment of differentially expressed genes between early vs. mid and mid vs. endpoint stage malignant cells, respectively. (F) GSEA confirmed upregulation of synaptic organization pathway in mid lesion compared to early lesion malignant cells. (G) Similarly, GSEA revealed further activation of synaptic organization pathway in enpoint stage compared to mid lesion malignant cells. (H) *Tubb* is key regulatory factor for synaptic organization pathway, and its expression gradually increases throughout the entire tumor progression process. Kaplan-Meier survival analysis showed that *Tubb* is a prognostic risk factor for patients with primary and recurrent gliomas. UMAP, uniform manifold approximation and projection. NBs, neuroblasts. NSCs, neural stem cells. OPCs, oligodendrocyte progenitor cells. TAPs, transient amplifying progenitors. GO: Gene Ontology. GSEA, Gene Set Enrichment Analysis. *Tubb, Tubulin Beta Class I***Table 1. Baseline characteristics of multifocal glioma patients.**

To further elucidate the mechanisms underlying glioma natural progression, we employed scRNA-seq to analyze molecular biological differences in mouse gliomas across distinct stages. Uniform manifold approximation and projection (UMAP) visualization in Fig. 5A revealed cellular clusters within early lesion, mid lesion, and endpoint stage gliomas, including choroid plexus cells, ependymal cells, immune cells, vascular endothelial cells, malignant cells, neuroblasts, neural stem cells/astrocytes, oligodendrocytes, oligodendrocyte progenitor cells (OPCs) and transient amplifying progenitors. These populations demonstrate the heterogeneity of the glioma microenvironment. We subsequently extracted malignant cells from each stage and annotated their distribution in the UMAP space (Fig. 5B). Using Monocle 3, we first analyzed the differentiation trajectory of malignant cells across stages and then validated the differentiation sequence through pseudotemporal inference (Fig. 5C).

Following data validation, we conducted differential gene expression and GO enrichment analyses between early lesions versus mid lesions and mid lesions versus endpoint stage malignant cells. Results in Fig. 5D show that differentially expressed genes between early and mid lesions were significantly enriched in synaptic organization and related regulatory pathways (Benjamini-Hochberg adjusted *p* < 0.05). GSEA confirmed significant upregulation of synaptic organization pathway in mid stage compared to early lesion malignant cells (Fig. 5F). Similarly, comparative analysis between mid lesion and endpoint stage revealed further activation of synaptic organization pathway in endpoint stage malignant cells (Fig. 5E, G). Based on malignant cell progression stages, we identified 42 sequentially and significantly (differential expression was considered statistically significant when the absolute value of Avg_log2 fold changes was ≥ 0.25 between comparison groups, with Benjamini-Hochberg adjusted *p* < 0.05) upregulated genes (Table 2), among which *Tubulin Beta 5 Class I (Tubb5, Tubb* in Homo sapiens) was key molecule involved in synaptic organization pathway. Using the mRNAseq_325 dataset from the CGGA, we evaluated the correlation between *Tubb* expression and patient prognosis. The results indicate that high expression of *Tubb* are associated with poor prognosis in both primary and recurrent gliomas (Fig. 5H).

**Table 2:** Genes exhibiting progressively elevated expression throughout glioma progression.

**Gene**	**Avg_log2 fold changes**		**Gene**	**Avg_log2 fold changes**	
	**Mid vs. Early**	**End vs. Mid**		**Mid vs. Early**	**End vs. Mid**
*Aldoa*	0.34	0.29	*Noct*	0.32	0.37
*Anp32b*	0.27	0.38	*Npm1*	0.30	0.46
*Aprt*	0.27	0.32	*Pebp1*	0.35	0.29
*Arl4a*	0.31	0.25	*Phlda1*	0.27	0.44
*Atf5*	0.33	0.28	*Rpl19*	0.31	0.26
*Capza2*	0.79	2.53	*Rpl30*	0.40	0.30
*Cct5*	0.39	0.32	*Rpl37*	0.29	0.30
*Cdkn2a*	0.98	0.34	*Rpl8*	0.42	0.44
*Eef1a1*	0.48	0.47	*Rps12*	0.44	0.33
*Eef1b2*	0.45	0.27	*Rps3*	0.58	0.26
*Eif3e*	0.27	0.33	*Rps4x*	0.57	0.28
*Eif5a*	0.30	0.28	*Rps9*	0.44	0.34
*Fkbp3*	0.28	0.32	*S100a10*	0.52	0.27
*H2afz*	0.58	0.43	*Timp1*	0.31	0.29
*Igfbp2*	0.36	0.40	*Tpm1*	0.28	0.54
*Iigp1*	0.71	0.36	*Tshz2*	0.40	0.36
*Lgals1*	0.42	0.90	*Tubb5*	0.44	0.31
*Lmo4*	0.29	0.27	*Ube2c*	0.40	0.52
*Met*	0.92	1.62	*Ube2s*	0.37	0.31
*Mif*	0.34	0.33	*Uchl1*	0.34	0.69
*Moxd1*	0.29	0.42	*Vim*	0.93	0.29

## 4 Discussion

This multimodal and cross-scale study provides novel insights into the patterns and mechanisms underlying glioma natural progression. By integrating advanced neuroimaging techniques and network theory in patients with multifocal gliomas, we systematically characterized macroscopic changes during tumor progression. Combined with longitudinal scRNA-seq analysis of mouse glioma samples, we further identified microscale molecular alterations and their mechanistic basis during glioma advancement. These findings delineate fundamental directional changes in glioma natural progression, elucidate potential mechanistic underpinnings, and may inform future therapeutic strategies targeting glioma-neuron interactions.

The progression of glioma is typically assessed by evaluating tumor cell proliferation, migration, invasion, and chemoresistance, which traditionally rely on invasive tissue sampling. In contrast, our study employs advanced neuroimaging techniques to noninvasively delineate the natural progression patterns of glioma. We discovered that gliomas develop higher FC with the cerebral cortex during progression and have more significantly related brain regions, indicating that FC enhancement between tumor and brain tissue occurs across widespread cortical areas. Further network analysis revealed increased degree centrality of gliomas within whole-brain networks, reflecting their heightened capacity to participate in signal transduction and information exchange throughout the brain. These findings suggest that gliomas are not merely isolated pathological entities within the nervous system, but rather exhibit parasitic-like characteristics by hijacking and exploiting the host's brain networks. This perspective aligns with recent advances in cancer neuroscience, which demonstrate that glioma cells secrete glutamate, Glypican-3, and Thrombospondin-1 to induce neuronal hyperexcitability and functional remodeling of neural circuits (Buckingham et al., 2011, Yu et al., Feb 2020, Krishna et al., May 2023). Conversely, neuronal activity promotes glioma progression through electrochemical communication via neuron-glioma synapses, potassium-induced currents, and the secretion of growth factors such as Neuroligin 3, Insulin-like Growth Factor 1, and Brain-derived Neurotrophic Factor (Venkatesh et al., 2015, Pan et al., Jun 2021, Venkatesh et al., 2017, Venkataramani et al., Sep 2019, Chen et al., Jun 2022). The glioma-induced potentiation of neuronal activity further amplifies activity-dependent regulation of tumor progression. Collectively, these mechanisms underscore how bidirectional interactions between the nervous system and gliomas drive disease progression. Our study expands this paradigm by demonstrating that glioma natural progression also reinforces the structural and functional integration of tumors with neural circuits.

The interaction mechanisms between glioma cells and neurons encompass synaptic connections, electrophysiological signaling, paracrine communication, metabolic, and neurotransmitter-related pathways (Wang et al., 2025). Our study revealed progressive activation of synaptic organization and associated regulatory pathways in glioma cells during natural progression, suggesting enhanced capacity to form synaptic structures with neurons. These microscale findings provide a mechanistic explanation for the macroscale enhancement of FC between gliomas and the cerebral cortex observed in our work. In alignment with this, Saritha et al. demonstrated that high-FC regions within glioblastoma exhibit significantly greater synaptic stability and synaptogenesis compared to low-FC regions, further supporting the influence of neuron-glioma synaptic interactions on macroscale FC (Krishna et al., May 2023). Moreover, our results are corroborated by previous studies investigating glioma progression through comparisons between primary and recurrent tumor samples. Frederick et al. identified significantly upregulated genes in recurrent *IDH*-wildtype gliomas and performed enrichment analysis, revealing increased stemness features and neuronal signaling activity (Varn et al., 2022). Similarly, Wu et al. compared different grades of *IDH*-mutant gliomas as well as primary and recurrent *IDH*-mutant gliomas, observing a gradual shift in tumor cell states from OPC-like to neural progenitor cell (NPC)-like phenotypes (Wu et al., Jan 2025). NPC-like glioma cells exhibit molecular and functional properties similar to normal NPCs and can interact with neurons via synaptic connections and calcium signaling (Venkataramani et al., 2022). Our study further revealed that the expression of the *Tubb* gene in malignant cells progressively increased during glioma progression. As a key molecule involved in synaptic organization pathways, *Tubb* not only associates with neuron-glioma synaptic interactions but also significantly impacts patient prognosis. *Tubb* encodes β-tubulin, which participates in microtubule formation (Miller et al., 2010). Although its role in gliomas remains poorly characterized, our findings suggest that *Tubb* may represent a novel therapeutic target for blocking glioma progression and improving clinical outcomes.

We did not observe significant differences in ALFF, fALFF, or ReHo values between glioma lesions, which may be attributed to shared pathological characteristics among multifocal tumor sites. ALFF and fALFF represent fMRI-derived metrics of voxel-wise neural activity intensity. Previous studies have demonstrated that high-grade gliomas exhibit significantly elevated ALFF/fALFF values compared to both low-grade gliomas and contralateral normal cortex (Gupta et al., Jun 2018), reflecting tumor-grade-dependent variations in angiogenesis, blood perfusion, and metabolic demand between neoplastic and normal tissues (Gevertz and Torquato, 2006, Kamba et al., Dec 2007, Patel et al., 2014). ReHo quantifies regional neural activity synchrony, which gliomas may disrupt through neurovascular uncoupling mechanisms (Agarwal et al., May 2017). The capacity for glioma-induced neurovascular uncoupling correlates with tumor grade, *IDH* mutation status, perfusion characteristics, and microvascular proliferation (Cai et al., Jul 2022, Englander et al., Jun 2018, Sun et al., Mar 2020, Bowden et al., Mar 2018), while the latter two features being intrinsically associated with tumor grading. Given that multifocal glioma lesions share clonal origin and typically maintain identical grading and IDH mutation status, this biological homogeneity explains the absence of interlesional ALFF/fALFF/ReHo variability. These findings underscore the critical advantage of studying glioma natural progression through multifocal cases rather than recurrent tumors, as long-term temozolomide treatment may induce hypermutation and malignant transformation in recurrent low-grade gliomas (Mathur et al., 2020).

Our study has several limitations. First, as a retrospective single-center imaging analysis with a relatively small sample size, the statistical power may be limited. Future multicenter studies with larger cohorts are required to validate our findings. Second, although we used contralateral homologous regions as controls, gliomas can exert remote effects through structural and functional networks, including alterations in the contralateral hemisphere. Consequently, the differences observed between tumor and control regions may partially underestimate the true impact of the tumor. Nevertheless, even under this relatively conservative control scheme, we detected significant between-group differences that remained robust after controlling for multiple technical confounds. This suggests that the observed changes primarily reflect genuine progression-related alterations rather than methodological bias alone. Future functional imaging studies in glioma could consider more refined control strategies, such as individualized expectation values derived from large-sample normative atlases or multimodal approaches that incorporate structural integrity (e.g., DTI) to identify truly unaffected regions. Additionally, although bioinformatic analysis of longitudinal mouse glioma samples revealed microscale mechanisms underlying glioma natural progression, future studies are warranted to validate these mechanistic insights in human glioma specimens through in vitro and in vivo experiments, given the inherent interspecies differences.

In conclusion, our study delineates both the macroscale patterns and microscale mechanisms underlying glioma natural progression. FC between tumors and the cerebral cortex, along with nodal topological properties of the tumors, may serve as noninvasive biomarkers for assessing glioma progression. Dynamic monitoring of these features could assist clinicians in predicting disease trajectory and informing therapeutic strategies. Furthermore, targeting the synaptogenesis process between glioma cells and neurons represents a promising therapeutic approach to halt glioma progression.

## CRediT authorship contribution statement

**Jiacheng Lai:** Writing – original draft, Visualization, Formal analysis, Data curation. **Yan Bai:** Writing – review & editing, Methodology, Data curation. **Hongbo Bao:** Writing – review & editing, Investigation. **Shuai Wu:** Writing – review & editing, Investigation. **Xinyu Wang:** Writing – review & editing, Investigation. **Xia Liang:** Writing – review & editing, Supervision, Methodology. **Peng Liang:** Writing – review & editing, Supervision, Resources, Project administration, Investigation, Conceptualization.

## Funding

This study has received funding supported by the National Natural Science Foundation of China (82572320), the Key Research and Development Program of Heilongjiang Province (2024ZX12C14), the Beijing Natural Science Foundation (JQ23040), and the Beijing Postdoctoral Foundation.

## Declaration of competing interest

The authors declare that they have no known competing financial interests or personal relationships that could have appeared to influence the work reported in this paper.

## Data Availability

Data will be made available on request.

## Appendix A Supplementary data

Supplementary Data 1

Supplementary Fig. 2 Differences in topological characteristics of tumor nodes in brain networks under sparsity thresholds.

Supplementary Fig. 3 Mean FD distribution and mean DVARS distribution.
