# Supplementary material for: Natural progression of glioma enhances functional connection with the cerebral cortex through synaptogenesis
Source: Neuroimage Clin. 2026 Jan 4;49:103942. doi: 10.1016/j.nicl.2026.103942 (PMC12814075; doi:10.1016/j.nicl.2026.103942)
Supplement: Supplementary Data 1 [file mmc1.doc]

**Supplementary Figure**

**
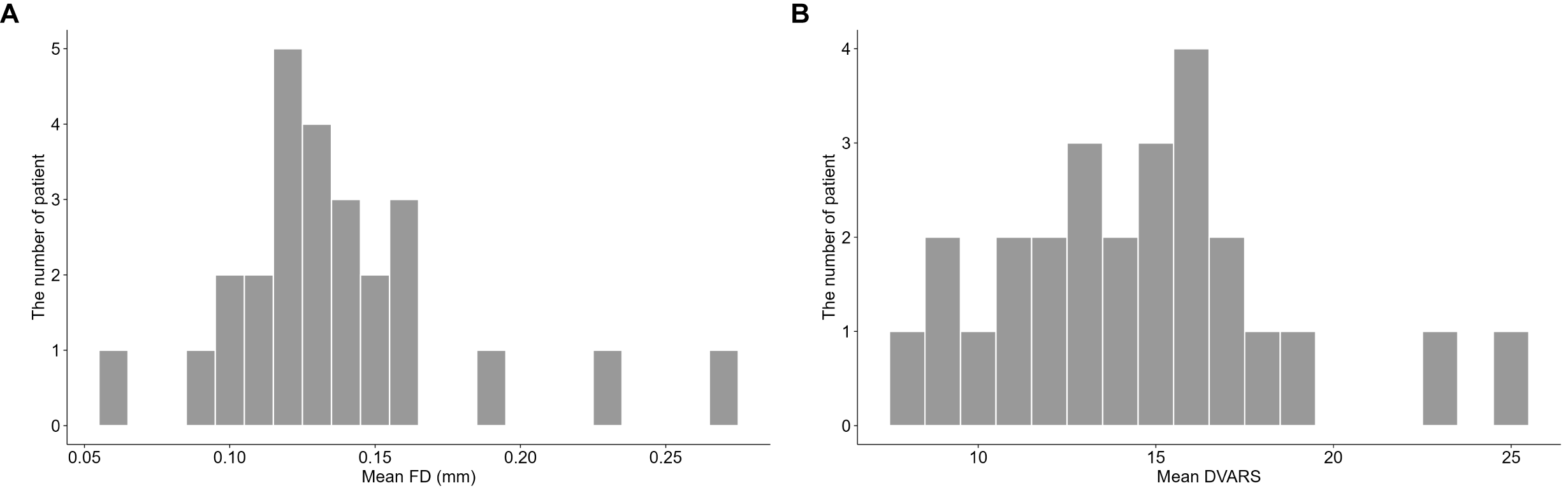
**

**Figure S1. Mean FD distribution (A) and mean DVARS distribution (B).**

**
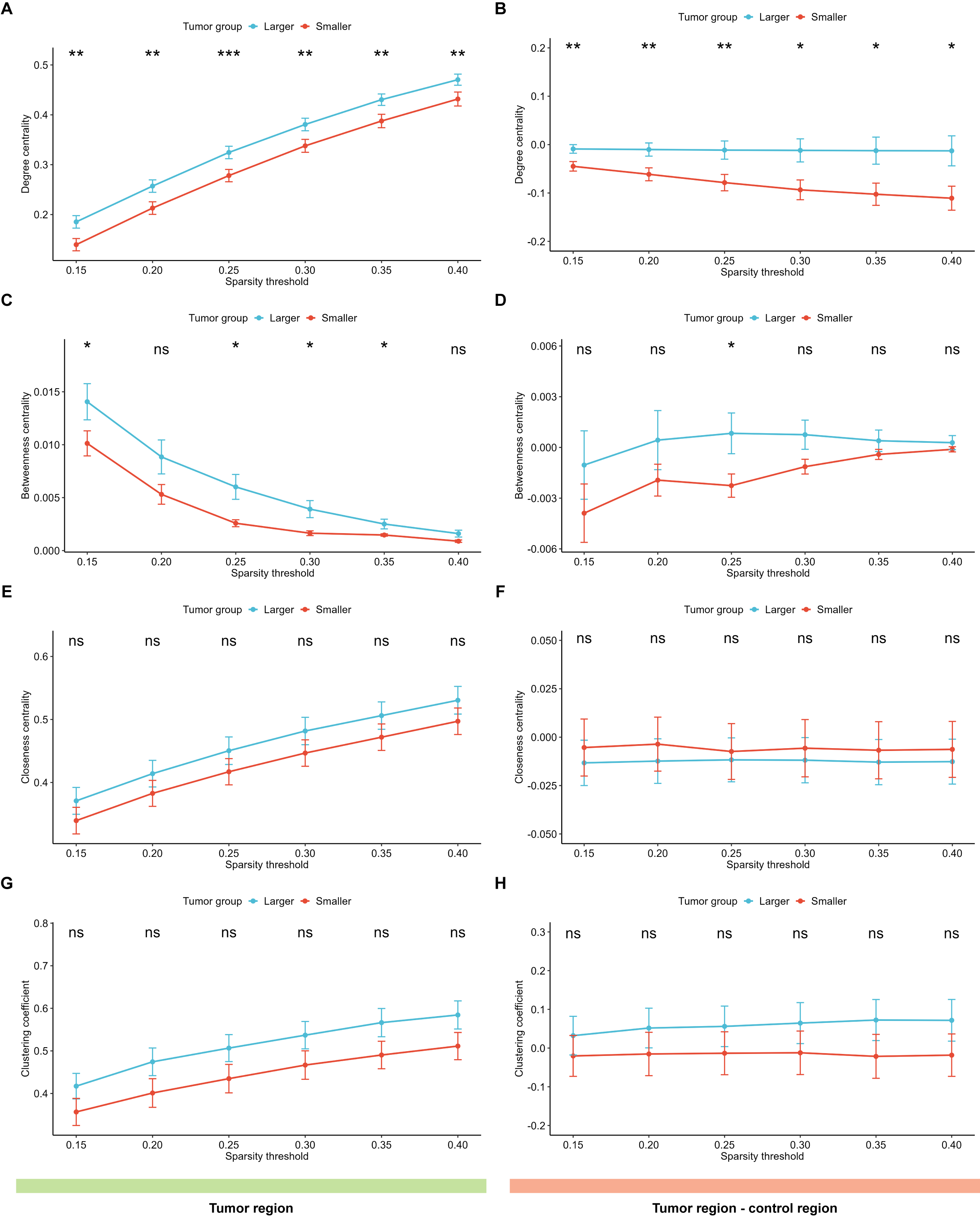
**

**Figure S2. Differences in topological characteristics of tumor nodes in brain networks under sparsity thresholds.** Paired comparisons were conducted between larger and smaller tumors across a sparsity range of 0.15–0.4 for differences in degree centrality (A), betweenness centrality (C), closeness centrality (E), and clustering coefficient (G). Subsequently, by subtracting the corresponding measures of the control region from those of the tumor region, the alterations in these network topological metrics in both tumor groups relative to the control region were evaluated (B, D, F, H).
