# Supplementary figures and images for: Natural progression of glioma enhances functional connection with the cerebral cortex through synaptogenesis
Source: Neuroimage Clin. 2026 Jan 4;49:103942. doi: 10.1016/j.nicl.2026.103942 (PMC12814075; doi:10.1016/j.nicl.2026.103942)

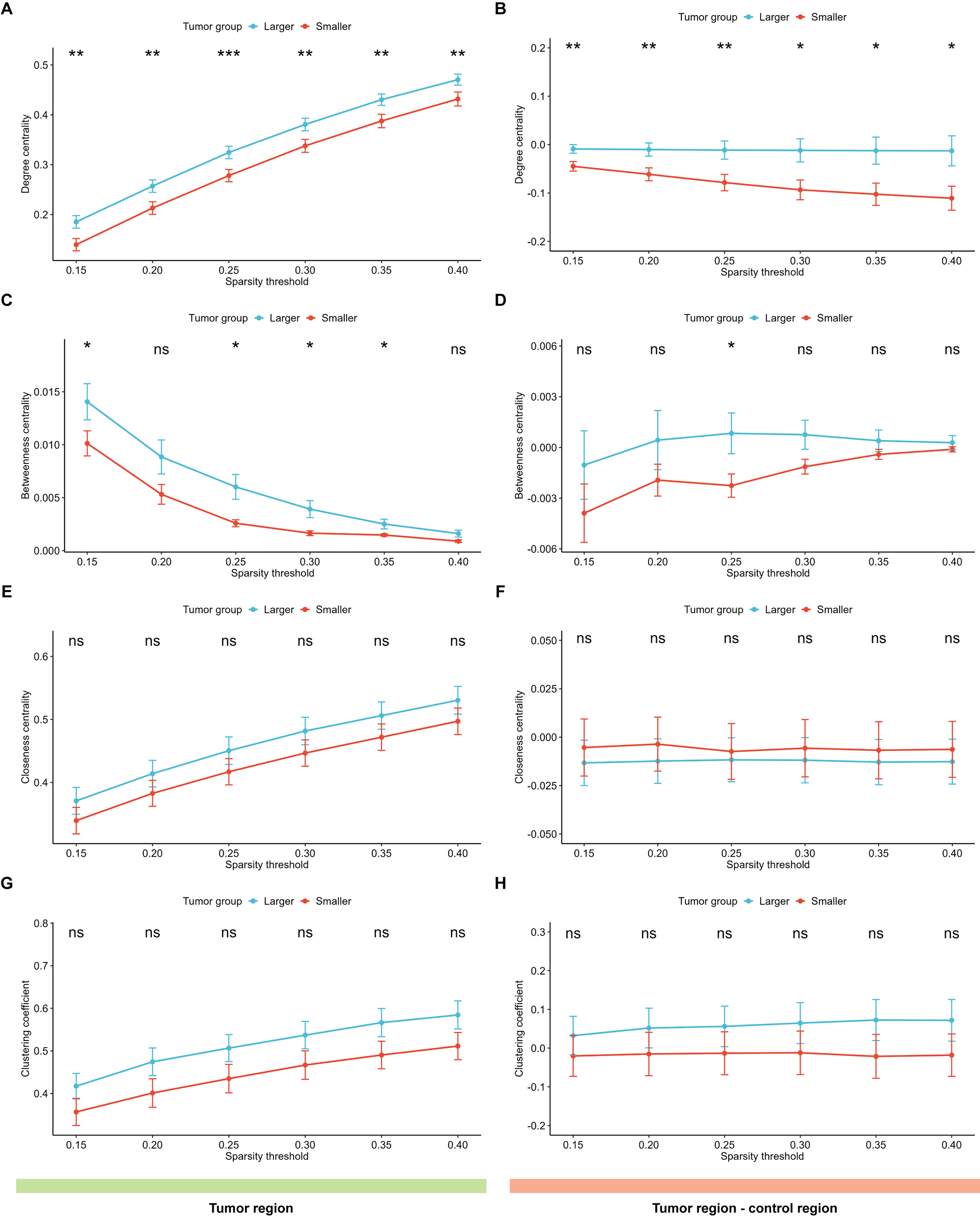

Supplement: Supplementary Fig. 2 — Differences in topological characteristics of tumor nodes in brain networks under sparsity thresholds. [file mmc2.jpg]

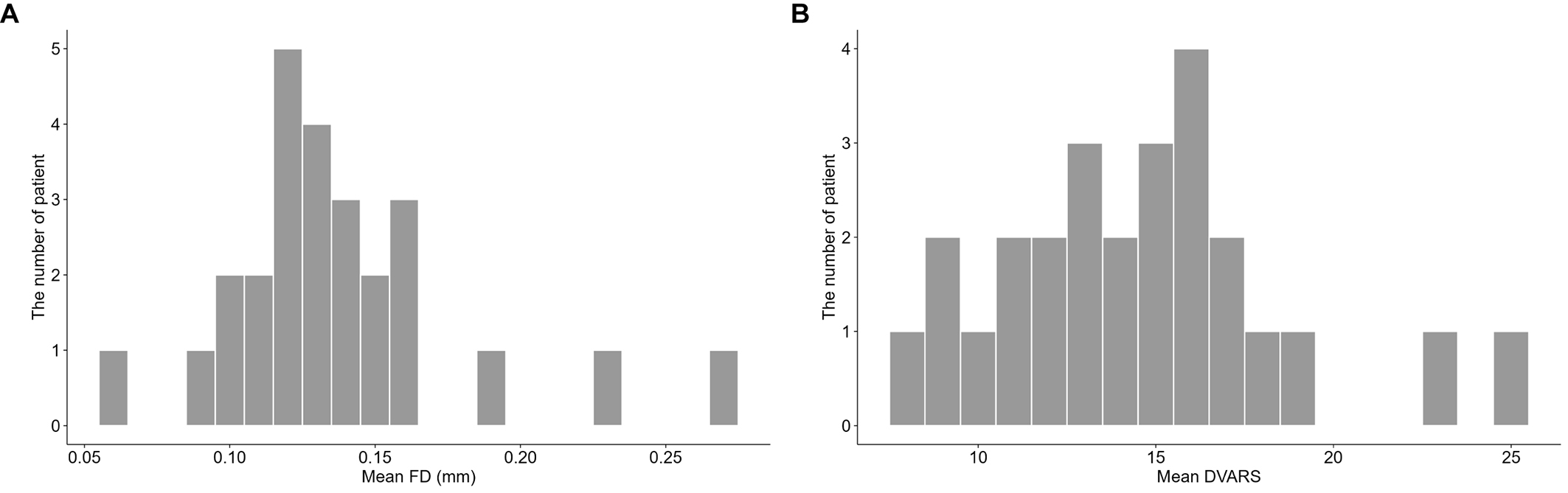

Supplement: Supplementary Fig. 3 — Mean FD distribution and mean DVARS distribution. [file mmc3.jpg]
